# Deep Learning-Assisted Detection and Classification of Thymoma Tumors in CT Scans

**DOI:** 10.3390/diagnostics15243191

**Published:** 2025-12-14

**Authors:** Murat Kılıç, Merve Bıyıklı, Salih Taha Alperen Özçelik, Hüseyin Üzen, Hüseyin Fırat

**Affiliations:** 1 Department of Thoracic Surgery, Faculty of Medicine, Inonu University, Malatya 44210, Türkiye; murat.kilic@inonu.edu.tr (M.K.); merve.biyikli@inonu.edu.tr (M.B.); 2 Department of Electrical and Electronics Engineering, Faculty of Engineering, Bingöl University, Bingöl 12000, Türkiye; sozcelik@bingol.edu.tr; 3 Department of Computer Engineering, Faculty of Engineering and Architecture, Bingol University, Bingöl 12000, Türkiye; huzen@bingol.edu.tr; 4 Department of Computer Engineering, Faculty of Engineering, Dicle University, Diyarbakır 21280, Türkiye

**Keywords:** thymoma disease, MLP-Mixer, VGG16, computed tomography

## Abstract

**Background/Objectives:** Thymoma is a rare epithelial neoplasm originating from the thymus gland, and its accurate detection and classification using computed tomography (CT) images remain diagnostically challenging due to subtle morphological similarities with other mediastinal pathologies. This study presents a deep learning (DL)-based model designed to improve diagnostic accuracy for both thymoma detection and subtype classification (benign vs. malignant). **Methods:** The proposed approach integrates a pre-trained VGG16 network for efficient feature extraction—capitalizing on its capacity to capture hierarchical spatial features—and an MLP-Mixer-based feature enhancement module, which effectively models both local and global feature dependencies without relying on conventional convolutional mechanisms. Additionally, customized preprocessing and post-processing methods are employed to enhance image quality and suppress redundant data. The model’s performance was evaluated on two classification tasks: distinguishing thymoma from healthy cases and discriminating between benign and malignant thymoma. Comparative analysis was conducted against state-of-the-art DL models including ResNet50, ResNet34, SEResNeXt50, InceptionResNetV2, MobileNetV2, VGG16, InceptionV3, and DenseNet121 using metrics such as F1 score, accuracy, recall, and precision. **Results:** The model proposed in this study obtained its best performance in thymoma vs. healthy classification, with an accuracy of 97.15% and F1 score of 80.99%. In the benign vs. malignant task, it attained an accuracy of 79.20% and an F1 score of 78.51%, outperforming all baseline methods. **Conclusions:** The integration of VGG16’s robust spatial feature extraction and the MLP-Mixer’s effective feature mixing demonstrates superior and balanced performance, highlighting the model’s potential for clinical decision support in thymoma diagnosis.

## 1. Introduction

The thymus gland, derived from the Greek word thymos meaning “spirit,” is a primary lymphoid organ that develops early in embryogenesis from the third pharyngeal pouch pair, migrating to the anterior mediastinum by the seventh week of gestation [1]. Located between the sternum and pericardium, it consists of two lobes with cortical and medullary regions. The thymus weighs about 10–15 g at birth, reaches its maximum size during infancy, and gradually involutes into fatty tissue after puberty; stress and other factors may cause reversible atrophy [2]. Functionally, it plays a crucial role in immune development by regulating T lymphocyte production, differentiation, and selection, and indirectly supporting B lymphocyte maturation [3,4,5,6].

Thymic epithelial cells can give rise to a variety of tumors, including thymomas—the most common primary anterior mediastinal neoplasm, representing roughly half of benign anterior mediastinal masses [7]. Thymomas are slow-growing, typically occur in adults aged 40–60, and show no clear gender or racial predilection. Approximately 30–50% of cases are associated with paraneoplastic syndromes, most often myasthenia gravis [8,9]. While some thymomas are asymptomatic, others cause symptoms due to local compression or paraneoplastic effects, such as dyspnea, cough, chest pain, superior vena cava syndrome, and hoarseness [9,10]. Because numerous lesions may arise in the anterior mediastinum—including thymic cysts, thymic carcinomas, germ cell tumors, lymphomas, and substernal goiters—diagnosis and evaluation require a multidisciplinary approach. Notably, about 59% of anterior mediastinal masses are malignant [9,11,12].

Computed tomography (CT) serves as a vital tool in the diagnosis and evaluation of these masses [13]. Thanks to its high resolution and superior soft-tissue contrast, CT can accurately depict the size, composition (solid or cystic), density, and relationship of the tumor to surrounding structures [9]. Contrast-enhanced CT is especially valuable for evaluating the tumor’s relationship with major mediastinal vessels and other adjacent structures. However, in patients with contrast allergies or renal dysfunction, magnetic resonance imaging (MRI) serves as a useful alternative that avoids ionizing radiation. The MRI provides excellent characterization of thymic lesions and their relationships to surrounding structures [14]. Definitive diagnosis of thymoma requires tissue sampling. The differential diagnosis includes substernal goiters, ectopic parathyroid tissue, germ cell tumors, lymphomas, thymic cysts, thymic carcinoid tumors, and, rarely, paragangliomas [9,15]. From a treatment standpoint, radical surgical resection remains the first-line and potentially curative approach for early-stage thymomas. In cases of locally advanced or unresectable thymomas, radiation therapy and chemotherapy serve as important adjunctive options [16]. The anatomical extent of thymic tumors, particularly their invasion into surrounding mediastinal structures, significantly impacts the clinical course and prognosis. Therefore, treatment planning must be conducted meticulously by a multidisciplinary team to ensure the best possible outcomes. In today’s healthcare settings, the increasing patient load and the growing demand for radiological imaging have led to a marked rise in the number of CT scans performed. Accurate and timely interpretation of these scans, especially in emergency departments and intensive care units, is critical for early diagnosis and treatment. However, under the burden of a heavy workload, there is a risk of misinterpretation, incomplete assessment, or delays in reporting, which can result in patient harm. Furthermore, changes in thymus size due to factors such as age and stress can make it challenging to differentiate normal thymic tissue from pathological lesions, increasing the likelihood of missed findings. Additionally, the dense vascular structures within the mediastinum can hinder tumor detection on non-contrast CT scans, allowing tumors to grow and invade surrounding tissues over time. This poses significant consequences for both patients and physicians. The gold standard for treating mediastinal tumors remains complete surgical resection. However, accurate diagnosis and early staging of thymic pathologies are essential for successful treatment. In this context, selecting the appropriate imaging modalities and detecting tumors at an early stage is of great importance for patient health.

In recent years, artificial intelligence (AI), particularly deep learning (DL) techniques, has achieved significant progress in the rapid and accurate analysis of imaging data within the healthcare sector. DL-based models have the potential to detect subtle details in radiological images and accurately classify pathological findings. As a result, they can support physicians under heavy workloads by reducing the risk of errors or incomplete interpretations. The effective use of AI in the diagnosis and staging of anterior mediastinal tumors, such as thymoma, could offer significant advantages for early diagnosis and accurate treatment planning. DL-based algorithms can precisely differentiate normal thymic tissue from pathological lesions and detect the presence of invasion, thereby enhancing clinical decision-making. Therefore, broader use of AI in healthcare presents an important opportunity to improve patient safety and enable early detection of diseases like thymoma.

### Studies in the Literature and Contributions of This Study

In recent times, several studies utilizing artificial intelligence have been published in the literature that concentrate on thymoma. Zhenguo Liu et al. [17] aimed to estimate the likelihood of myasthenia gravis in thymoma patients by utilizing a 3D DenseNet method that was trained with preoperative CT scans. Their dataset included 230 thymoma cases, with 182 (81 diagnosed with myasthenia gravis, 101 without myasthenia gravis) designated for training, while 48 additional external cases were used for external testing to assess the model’s generalizability. Alongside the 3D DenseNet, five radiomic models—MLP, logistic regression, SVM, XGBoost, and random forest—were also developed. Moreover, they proposed a multi-model framework that combined machine learning with semantic CT image features. The 3D DenseNet reached an accuracy of 0.724, whereas the integrated multi-model approach achieved a better accuracy of 0.790. The external validation also demonstrated the effectiveness of the DL-based multi-model approach, achieving an accuracy of 0.732. Liu et al. [18] suggested a 3D DL model using arterial phase CT images and clinical data to stratify thymoma risk. The model, based on transfer learning with ResNet50, was trained on 147 patient scans and outperformed both a radiomic model and a 2D deep model. It achieved AUCs of 0.998 (training) and 0.893 (testing), demonstrating superior accuracy in tumor segmentation and risk classification, supporting better clinical decision-making. Yang et al. [19] utilized CT scans from 174 patients with thymoma to create a deep learning-based system aimed at preoperative staging, focusing on differentiating Masaoka-Koga stage I from stage II thymomas. Two chest radiologists independently reviewed the images, and statistical analysis revealed imaging characteristics that varied between the stages. They trained a 3D-DenseNet DL model for stage classification, employing two distinct techniques for labeling the regions of interest (ROI): segmentation and bounding boxes. The findings demonstrated that conventional CT features provided limited accuracy for staging (AUC = 0.639), whereas the 3D-DenseNet model showed notable improvement, reaching an AUC of 0.773. Additionally, ROI labeling based on segmentation outperformed the bounding box method (AUC 0.773 compared to 0.722). Mehmet et al. [9] proposed a transformer-based DL methodology for the detection of thymoma, using patient image data from Fırat University’s Department of Thoracic Surgery. The dataset included images with and without thymoma. The method involved preprocessing images to focus on the region of interest, training four transformer models (ViT, Swin Transformer, MaxViT, and DeiT3), and extracting features from these models. Feature sets from the best-performing models were then combined and refined using the Minimum Redundancy Maximum Relevance feature selection technique. Finally, a Support Vector Machine classifier was employed to classify the images. The results were impressive, with the feature selection approach achieving 100% accuracy, and a cross-validation analysis showing 99.22% accuracy, highlighting the high potential of this transformer-based model for reliable thymoma detection.

Recently, only a few studies have focused on the classification and detection of thymoma disease, with most existing literature relying on conventional DL techniques and traditional machine learning approaches. To address this gap, our study introduces an MLP-Mixer-Based Feature Enhancement approach—a modern DL technique—to identify tumors in lung CT scans, replacing the conventional DL techniques typically used. While hybrid deep learning methods that combine CNNs with attention mechanisms or transformer-based models (e.g., ViT, Swin Transformer) have been explored in related tumor detection and classification tasks, to the best of our knowledge, no prior work has specifically combined VGG16 for feature extraction with an MLP-Mixer for feature enhancement in the context of thymoma. Technically, our approach differs by uniting VGG16’s strong hierarchical spatial feature extraction with the MLP-Mixer’s ability to capture both global and local dependencies without relying on convolution or self-attention. This synergy provides an effective solution to the challenges of limited data and subtle morphological differences in thymoma CT scans. From a therapeutic and clinical perspective, existing works often focus solely on either thymoma detection or staging. In contrast, our study addresses both thymoma—healthy classification and benign—malignant subtype discrimination, which are highly relevant for therapeutic decision-making and prognosis. Thus, our approach offers a more comprehensive diagnostic support tool than those reported in the literature.

In this study, we propose a VGG16–MLP-Mixer hybrid model for the automated detection and classification of thymoma tumors from lung CT scans. Unlike conventional CNN-based or transformer-based methods, the proposed approach introduces a feature enhancement strategy that bridges convolutional feature extraction with MLP-based global–local dependency modeling. The model is composed of five main stages: preprocessing, VGG16-based feature extraction, feature tokenization, MLP-Mixer-based feature enhancement, and final classification. This architecture provides a balanced integration of spatial detail and contextual reasoning, offering a technically distinct alternative to existing deep learning methods.

The main technical innovations and contributions of this study are as follows:

- The proposed model introduces a targeted mediastinal cropping and multi-slice fusion strategy to improve data relevance and reduce noise. By isolating the mediastinal region—where thymomas typically appear—and fusing adjacent CT slices into a three-channel composite image, the model captures inter-slice continuity and spatial context often overlooked in conventional 2D CNN pipelines. This preprocessing design forms the foundation for extracting anatomically meaningful representations, directly improving diagnostic sensitivity.
- Instead of using the MLP-Mixer as an independent classifier, this study proposes a feature-level fusion mechanism where features extracted from the pre-trained VGG16 are tokenized and refined by the MLP-Mixer. This integration enables the MLP-Mixer to act as a global refinement layer, enhancing the contextual relationships within CNN-derived feature maps. Such a sequential CNN–MLP combination has not been widely explored in medical CT analysis, making it a key technical innovation of this study.
- While the MLP-Mixer architecture was originally developed for natural image classification, its application here is adapted to capture both local spatial variations and global inter-channel dependencies in CT data. Through the Token-Mixer and Channel-Mixer layers, the model explicitly learns long-range dependencies and inter-feature relationships that CNNs alone cannot represent effectively. This design enhances the discriminative power of the feature space, improving the separation between thymoma subtypes.
- Leveraging VGG16’s pre-trained hierarchical representations ensures robust extraction of low- and mid-level features, mitigating overfitting. The MLP-Mixer then performs adaptive feature enhancement, allowing the model to generalize well even with restricted CT data availability—a common limitation in medical imaging tasks.
- Instead of relying on single-slice predictions, the model aggregates results across multiple CT segments to produce a unified diagnostic decision for tumor presence and subtype (benign or malignant). This aggregation step reduces prediction variance and increases clinical reliability, demonstrating the practical diagnostic value of the proposed framework.

In summary, the technical novelty of this study lies in the cross-domain integration of CNN and MLP-Mixer architectures through a tokenized feature enhancement pipeline, combined with a domain-specific preprocessing strategy tailored for thymoma CT classification. This hybrid approach offers a new direction for combining convolutional and MLP-based representations in medical image analysis.

The rest of the manuscript is structured as follows. In Section 2, a comprehensive description of the proposed model and its components is provided. Section 3 covers the dataset, the hyperparameters applied in the research, the evaluation criteria, and the findings from the conducted experiments. Lastly, Section 4 summarizes the conclusions drawn and outlines the planned future prospect.

## 2. Proposed Model

Computed Tomography (CT) images possess a three-dimensional structure along the x, y, and z axes, and specialist physicians typically examine these images slice by slice to make diagnostic assessments based on the entire CT dataset. However, this manual process is time-consuming and cognitively demanding, often requiring the analysis of hundreds of slices per patient. In recent years, the advancement of artificial intelligence technologies has made it possible to automate such diagnostic tasks, enabling faster and more consistent decision-making.

**Figure 1:**
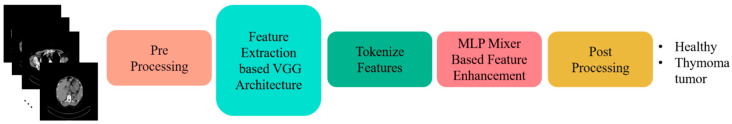
Pre-trained MLP-Mixer model.

In this context, the present study proposes an MLP-Mixer-Based Feature Enhancement method for the detection of thymoma tumors in lung CT images. The proposed model operates through five sequential stages (Figure 1). First, CT images undergo an anatomically guided preprocessing step to ensure that only the relevant mediastinal region is analyzed, thereby reducing irrelevant information. Next, the VGG16 architecture is employed as a pre-trained feature extractor to generate low- and mid-level spatial representations from the preprocessed CT images. These extracted feature maps are then tokenized—transformed into a structured sequence of non-overlapping feature tokens—allowing them to be processed within the MLP-Mixer-based feature enhancement block. Within this block, two distinct submodules, the Token-Mixer and Channel-Mixer, independently operate across spatial and channel dimensions using fully connected MLP layers instead of convolutional kernels or attention matrices. The Token-Mixer enables cross-token information exchange to model long-range spatial dependencies, while the Channel-Mixer refines inter-feature relationships, enhancing the discriminative quality of the learned representations. The enhanced features are then classified using a softmax classifier, and finally, a post-processing stage consolidates these predictions to determine whether the CT image indicates the presence of a thymoma tumor.

From a technical standpoint, the primary innovation of this study lies in the hybrid integration of convolutional and MLP-based architectures through a tokenized feature enhancement mechanism. Unlike conventional CNN models that rely solely on localized convolutional filters, or transformer-based models that depend on computationally expensive self-attention, the proposed approach employs the MLP-Mixer as a lightweight and fully feedforward alternative to capture both global and local dependencies. By converting the spatially encoded VGG16 feature maps into tokens and refining them through the MLP-Mixer’s dual-axis mixing operations, the model achieves efficient global contextual learning without the quadratic complexity of attention mechanisms. This design not only enhances feature expressiveness but also improves generalization in limited-data conditions, which is a critical challenge in medical imaging. Furthermore, the anatomically guided preprocessing and multi-stage integration pipeline make the proposed model both computationally efficient and clinically interpretable, representing a novel and technically distinctive contribution to CT-based tumor detection research.

The study was approved by the Scientific Research Ethics Committee of Inonu University Faculty of Health Sciences (Date: 30 April 2025, Session No: 2, Decision No: 2025/7030). Patient consent was waived due to the retrospective nature of the study.

### 2.1. Pre-Processing

A lung CT image typically has high resolution and contains numerous slices. Moreover, according to our research in the literature, there are no comprehensive thymoma datasets available. Therefore, the available data is limited. Due to this data limitation, a two-stage preprocessing step was designed to center on the likely location of the tumor and to use the data more effectively.

**Figure 2:**
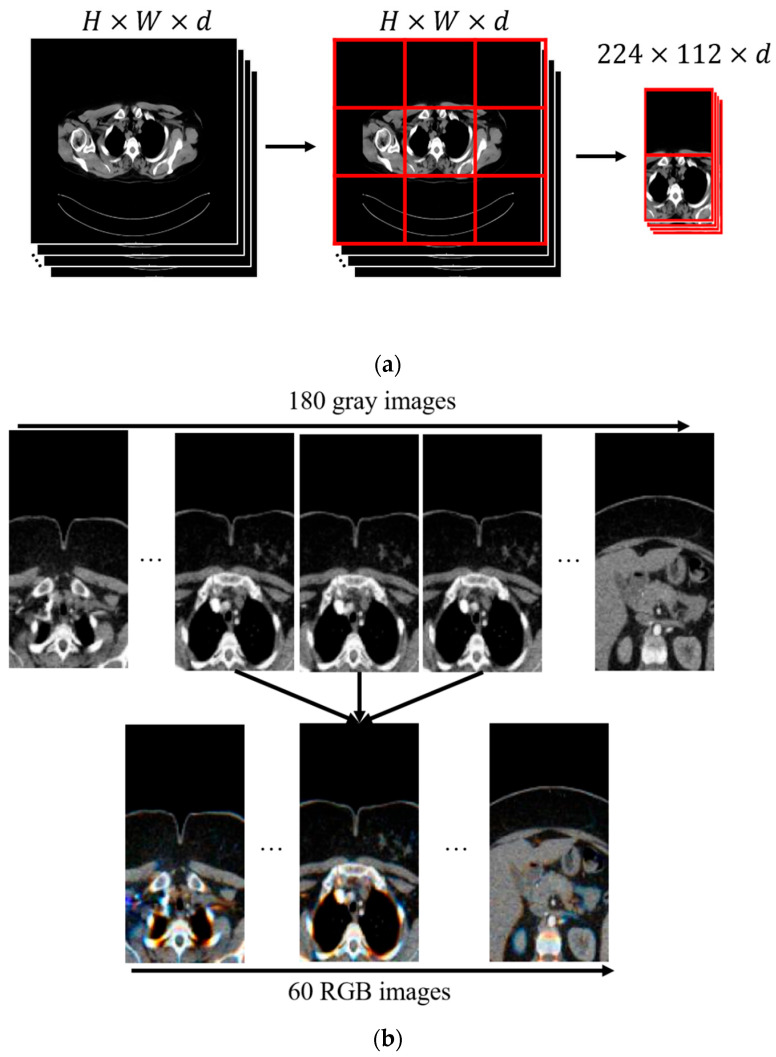
Pre-processing stage of thymoma images. (**a**) Mediastinal region cropping using a 3 × 3 grid; (**b**) CT slice stacking.

In the proposed preprocessing step, the first stage involves cropping the image based on the possible anatomical location where the tumor (thymoma) is commonly observed. The thymoma tumor typically occurs in the mediastinal region, that is, between the lungs within the thoracic cavity. Based on this, as shown in Figure 2a, the CT image is divided into a 3 × 3 grid. As a result of this division, the images in the first and second rows of the second column are cropped and selected for analysis. This approach directly focuses the model’s attention on areas where tumor formation is most likely, thereby increasing data efficiency. In this way, the limited data is utilized in the most efficient manner, and the diagnostic sensitivity of the model is also significantly improved. The second step in preprocessing involves reducing the number of CT slices (Figure 2b). Here, direct resizing was avoided because it could result in the loss of the limited number of tumor-containing slices. Additionally, since there is generally a high degree of similarity between slices, anomalies such as tumors can often be detected from the differences between them. Therefore, to reduce the data without losing details between slices, three consecutive grayscale slices were combined to create a three-channel (RGB-like) image. In this way, the number of slices is reduced without data loss, while also obtaining an input size suitable for the pre-trained VGG network architecture.

### 2.2. MLP-Mixer Based Feature Enhancement

**Figure 3:**
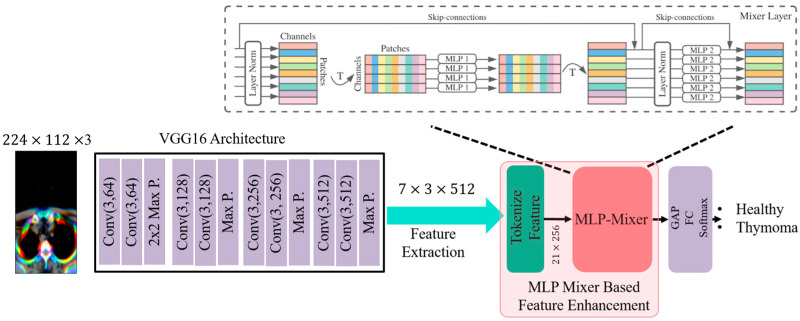
Proposed pre-trained VGG16-based MLP-Mixer model.

The MLP-Mixer architecture [20] is a deep learning model used in image processing that consists solely of multilayer perceptrons (MLPs). Unlike ViT [21] and ConvMixer [22] models, the MLP-Mixer does not include a self-attention mechanism or convolutional layers with large kernels (such as 3 × 3 or wider filters). Instead, it applies fully connected layers, called MLPs, to each patch of the input. Additionally, the MLP-Mixer model is designed to capture relationships both between patches and within the features of each patch. This allows the MLP-Mixer to extract both global and local features from an image. However, since the MLP-Mixer relies heavily on fully connected layers, it requires a large and powerful dataset. Datasets used for tumor detection typically contain a limited number of samples. Particularly in problems with high data variability, such as thymoma, it is quite difficult to form a strong dataset. Therefore, the MLP-Mixer model either fails to learn the problem effectively or suffers from overfitting when trained on a limited dataset. To address this issue, this study proposes an MLP-Mixer model supported by features extracted from a pre-trained VGG16 architecture. The model proposed in this study is illustrated in Figure 3.

**Figure 4:**
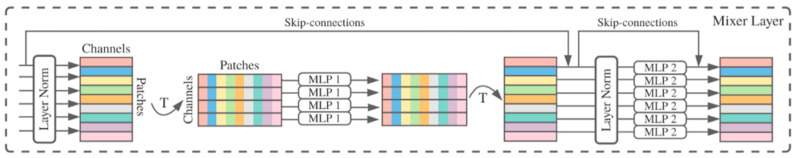
MLP-Mixer model [20].

As shown in Figure 3, the proposed model obtains a feature map with dimensions 7 × 3 × 512 in the final layer of the VGG architecture. In order to feed this feature map into the MLP-Mixer model, it needs to be tokenized. The tokenization process is carried out using a reshape layer. At the end of this process, a 21 × 512 feature output is obtained, and a 256-neuron MLP layer is applied to this output to generate 21 tokens. Each token contains 256 features, resulting in a token-feature matrix of size 21 × 256. These feature vectors are then passed to the MLP-Mixer model. The MLP-Mixer model is shown in Figure 4.

The MLP-Mixer model consists of two types of Mixer layers: the Token-Mixer and the Channel-Mixer. The Token-Mixer focuses on a feature across tokens, relating each token to the others to obtain global features. In this way, it enables the exchange of information between different tokens. To achieve this, a transpose operation is first applied to the input x to obtain the feature $x^{T}$. This feature is then passed through an MLP layer, which sequentially applies normalization, a fully connected layer, GELU (Gaussian Error Linear Unit) [23], and another fully connected layer. The weights of the fully connected layers applied here are shared across all token channels. The formalization of the MLP model is shown in Equation (1).

(1) $$\mathrm{MLP}\left(a_{i}\right)=W_{2}\left(\sigma \left(W_{1}\left(\mathrm{DN}\left(a_{i}\right)\right)\right)\right)\;\mathrm{for}\;i=1\ldots C\;$$

In Equation (1), a represents the input to the MLP block and has dimensions S × C. In this dimension representation, S denotes the number of tokens and C denotes the number of features contained within each token. Additionally, $a_{i}$ represents the *i*-th token of the input feature a. $W_{1}$ and $W_{2}$ represent the fully connected layers applied within the MLP block. The weights of the fully connected layers are shared for each token, meaning that the number of trainable parameters is the same for all tokens. The MLP model is similarly applied in both the Token-Mixer and the Channel-Mixer models. However, in the Token-Mixer model, the operation is based on the relationship between tokens, and therefore the input is transposed. This swaps the roles of features and tokens. The formalization of the Token-Mixer model is given in Equation (2).

(2) $$x_{\mathrm{token}\;\mathrm{mixer}}={\left(\mathrm{MLP}\left(x^{T}\right)\right)}^{T}+x$$

The MLP($x^{T}$) equation applied here relates the features of the same channel across all tokens to extract global features. To achieve this, the feature x is first transposed to swap the roles of tokens and features. Then, an MLP layer is applied. In this way, traditional channel-based operations are carried out in the spatial domain. At the final step of the Token-Mixer, the feature map obtained from MLP($x^{T}$) is transposed back to its original form. Finally, the element-wise sum of the input feature is computed. As a result, the output feature $x_{\mathrm{token}\;\mathrm{mixer}}$ is obtained.

As seen in Figure 4, in the second stage of the MLP-Mixer, the feature map $x_{\mathrm{token}\;\mathrm{mixer}}$ is transferred to the Channel-Mixer layer. Unlike the Token-Mixer layer, the Channel-Mixer layer applies the MLP layer along the channel dimension. This enables it to relate the features within each token. The Channel-Mixer layer is formalized in Equation (3).

(3) $$x_{\text{Channel-mixer}}=\mathrm{MLP}\left(x_{\mathrm{token}\;\mathrm{mixer}}\right)\;+x_{\mathrm{token}\;\mathrm{mixer}}$$

In Equation (3), $x_{\text{Channel-mixer}}$ represents the output, and this output is obtained by using $x_{\mathrm{token}\;\mathrm{mixer}}\;$ as the input. As a result, the input in the MLP-Mixer model facilitates information exchange both across channels and between tokens. Additionally, this process is carried out in two separate parts, significantly reducing the computational cost. In the proposed model, the MLP-Mixer is applied twice to a feature map of size 16 × 128 to obtain the final feature map for classification. For the classification process, the final feature map is first subjected to a global average pooling (GAP) layer. Following this layer, a fully connected layer with 2 neurons is added, and finally, a softmax layer is used to produce the classification prediction output.

### 2.3. Post-Processing

After applying the processes described above, the CT image patches are classified as either “healthy” or “tyhmoma tumor.” Following this step, all patch results for each CT scan need to be combined to reach a final decision for the corresponding CT image. For this purpose, the following algorithm is applied:

1. Each CT image consists of pre-divided patches.
2. All patches are analyzed individually, and each one receives a label of “thymoma” or “healthy” from the classifier.
3. The results are stored in an array.
4. If any patch in the array is classified as “thymoma”, the entire CT image is labeled as “thymoma”.
5. Otherwise, the CT image is labeled as “healthy.”

With this method, it is possible to reliably detect whether a CT image from a patient contains a thymoma lesion. Consequently, the model provides a clinically meaningful and practical final evaluation.

## 3. Analysis of Experimental Results

### 3.1. Dataset

**Figure 5:**
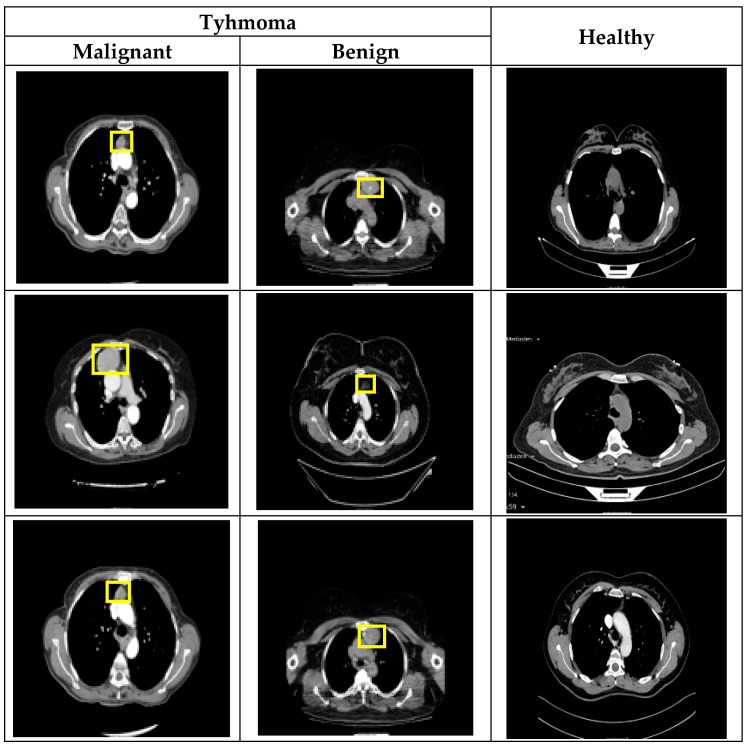
Example CT images from the dataset.

The CT image dataset used in this study was obtained from the Department of Thoracic Surgery, Turgut Ozal Medical Center, Inonu University. A total of 198 patients were included in the study, consisting of 120 healthy individuals and 78 thymoma patients. Among the thymoma patients, 46 were diagnosed with benign thymoma and 32 with malignant thymoma. Before preprocessing, the dataset contained 31,685 CT slices from healthy individuals, 576 slices from malignant thymoma cases, and 602 slices from benign thymoma cases. A preprocessing pipeline, including normalization, slice selection, and resizing, was applied to ensure consistent image quality. After this stage, the number of usable slices was reduced to 10,383 for healthy individuals, 132 for malignant thymoma, and 306 for benign thymoma. All CT images were anonymized prior to analysis. Preprocessing was performed at the slice level to remove low-quality frames and standardize the images for use in the deep learning models. A patient-based 5-fold cross-validation strategy was used to ensure reliable and independent evaluation of model performance. In this approach, patient identity—not individual slices—was used to separate training and testing sets, preventing any slices from the same patient from appearing in both subsets within a fold. In each fold, approximately 80% of the patients (around 158 individuals) were assigned to the training set, while the remaining 20% (around 40 individuals) formed the testing set. The folds were constructed to maintain the overall patient distribution as closely as possible, including 120 healthy patients, 46 benign thymoma patients, and 32 malignant thymoma patients. This ensured that each fold represented the general structure of the full dataset. Example CT images from the dataset are presented in Figure 5.

The study employed a two-stage classification approach. In the first stage, the model was trained to distinguish healthy individuals from thymoma patients. For this purpose, all healthy CT slices were labeled as “healthy,” while all slices belonging to thymoma patients (benign and malignant combined) were labeled as “thymoma.” The model was trained using this binary labeling scheme to identify whether a given CT image originated from a healthy subject or a patient diagnosed with thymoma. In the second stage, an additional classification task was performed to differentiate between benign and malignant thymoma cases. For this step, only the CT images from thymoma patients were used. The slices were labeled according to their respective diagnoses—“benign” or “malignant”—and the classification model was trained again using this two-class labeling structure. This stage enabled the evaluation of the model’s ability to identify the malignancy level of thymoma based on CT images. This sequential strategy allowed the proposed system to first detect the presence of thymoma and subsequently evaluate its malignancy, providing a clinically meaningful diagnostic workflow.

### 3.2. Criteria for Evaluation

Several evaluation metrics were used to assess the performance of the proposed model in detecting thymoma tumors from CT images. These metrics include: Accuracy, precision, recall, and F1 score. Each metric emphasizes different aspects of model performance, which is crucial in medical image analysis, where both false positives and false negatives can have significant clinical implications. The accuracy measures the proportion of correct predictions (both positive and negative) out of the total predictions made. In the context of medical diagnosis, accuracy provides a general sense of how often the model correctly identifies whether a CT image shows a thymoma tumor or not. Accuracy is calculated as in Equation (4):

(4) $$\mathrm{Accuracy}\;=\;\frac{\mathrm{TP}+\mathrm{TN}}{\mathrm{TP}+\mathrm{TN}+\mathrm{FP}+\mathrm{FN}}$$

While accuracy offers a quick overview of the model’s overall correctness, it may not be sufficient alone in medical imaging tasks due to potential class imbalances (e.g., more non-tumor cases than tumor cases). Therefore, additional metrics are critical. The precision indicates the proportion of cases predicted as positive (thymoma tumor present) that are actually positive. In the detection of thymoma tumors, high precision means fewer incorrect positive diagnoses, reducing the likelihood of unnecessary treatments or further interventions. It is defined as in Equation (5):

(5) $$\mathrm{Precision}\;=\;\frac{\mathrm{TP}}{\mathrm{TP}\;+\;\mathrm{FP}}$$

The recall (also known as sensitivity) measures the model’s ability to correctly identify actual positive cases. In this context, recall reflects how well the model detects true thymoma tumors in CT images. High recall minimizes the risk of missing tumors, ensuring that patients with thymoma are properly identified and treated. It is defined as in Equation (6):

(6) $$\mathrm{Recall}\;\mathrm{or}\;\mathrm{Sensitivity}\;=\;\frac{\mathrm{TP}}{\mathrm{TP}\;+\;\mathrm{FN}}$$

The F1 Score is the harmonic mean of precision and recall, providing a single metric that balances the trade-offs between false positives and false negatives. This balance is crucial in medical imaging applications like thymoma detection, where both overdiagnosis and missed cases carry significant risks. A high F1 score indicates that the model performs well in both correctly identifying thymoma tumors and minimizing incorrect tumor predictions, making it a reliable metric for this medical diagnosis task. The F1 score is computed as in Equation (7):

(7) $$F1\;\mathrm{Score}\;=\;2\;\times \;\frac{\mathrm{Precision}\;\times \;\mathrm{Recall}}{\mathrm{Precision}\;+\;\mathrm{Recall}}$$

In the above equations, the terms TP, TN, FP, and FN are derived from the confusion matrix: True positives (TP) represent cases where the model correctly identifies the presence of a thymoma tumor in a CT image. True negatives (TN) represent cases where the model correctly identifies the absence of a thymoma tumor. False positives (FP) occur when the model incorrectly predicts a thymoma tumor in an image where none exists, potentially leading to unnecessary diagnostic procedures or patient distress. False negatives (FN) occur when the model fails to detect an existing thymoma tumor, which may delay critical treatment. These metrics together provide a comprehensive evaluation framework to rigorously assess the clinical viability of the proposed model for thymoma tumor detection using CT imaging.

### 3.3. Setting of Hyperparameters

In the experimental studies, the Python programming language (version 3.9) was used along with the Keras (version 2.10) and TensorFlow (version 2.10) libraries. The training and testing of the proposed models were carried out on a computer equipped with an Intel i7 processor, 128 GB of RAM, and an RTX 4090 GPU. The hyperparameters used for training the model were as follows: a batch size of 64, a learning rate of 0.0001, and 100 epochs. The Adam optimization algorithm was employed. The input image dimensions were set to 224 × 112 × 3, and the Categorical Cross-Entropy loss function was used during model training. All experimental evaluations were performed using a 5-fold cross-validation scheme to ensure a reliable assessment of the model’s robustness and generalization capability. This approach was adopted to make efficient use of the available data, given the relatively limited number of patients in the dataset. By dividing the data into five subsets and iteratively using four folds for training and one for validation, the model’s performance could be evaluated across multiple data partitions, thereby reducing the likelihood of bias associated with a single train–test split and providing a more stable estimate of the model’s true performance. Additionally, performance metrics, including accuracy, precision, recall, and F1-score, were calculated for each fold, and the final results were obtained by averaging the performance across all five folds. Additionally, early stopping was not employed in the experimental studies. Instead, each model was trained for a fixed total of 100 epochs to maintain consistency across folds and to enable a uniform comparison of performance throughout the 5-fold cross-validation procedure.

### 3.4. Results of Experimental Studies

#### 3.4.1. Thymoma/Healthy Classification Results

In the experimental studies, the primary goal was to classify CT images as either thymoma or healthy. Accordingly, the proposed model was compared with various DL-based methods from the literature, including Vision Transformer (ViT) [21], SEResNeXt50 [24], MobileNetV2 [25], InceptionV3 [26], InceptionResNetV2 [27], DenseNet121 [28], VGG16 [29], ResNet34 [30], ResNet50 [30], and Swin Transformer [31]. The classification results are provided in Table 1. The metrics used for evaluation include accuracy, F1 score, recall, and precision, which together provide a well-rounded view of each model’s classification capability. Across the board, all models demonstrate high classification accuracy, with scores typically exceeding 96%. Notably, the proposed model outperforms all baseline models with an accuracy of 97.15%, highlighting its superior ability to accurately identify both thymoma and healthy cases. The proposed model is closely followed by InceptionResNetV2 and ResNet34, which achieve accuracies of 97.02% and 97.07%, respectively, demonstrating their robustness in this classification task. When it comes to the F1 score, which balances recall and precision, the proposed model again leads significantly with a score of 80.99%, while the second-best model, DenseNet121, achieves 77.51%. This difference indicates that the proposed model achieves a better balance between detecting thymoma cases (recall) and minimizing false positives (precision). In terms of the recall, which is particularly critical in medical diagnostics to avoid missing actual cases of thymoma, ResNet34 exhibits the highest recall at 85.99%. However, despite its high recall, its relatively low precision (71.72%) pulls down its F1 score. The proposed model follows with a recall of 82.56%, indicating it captures the majority of thymoma cases while maintaining strong overall performance in the other metrics. The precision, representing the proportion of true positive identifications out of all positive predictions, is again highest in the proposed model with 79.92%, signifying fewer false positives. This is crucial in medical settings to prevent unnecessary alarm or treatment in healthy individuals. In conclusion, while several individual models perform well in a few metrics, the proposed model demonstrates a strong and consistent performance across all evaluation criteria, making it the most balanced and reliable model for the thymoma vs. healthy CT image classification task presented in this study.

**Figure 6:**
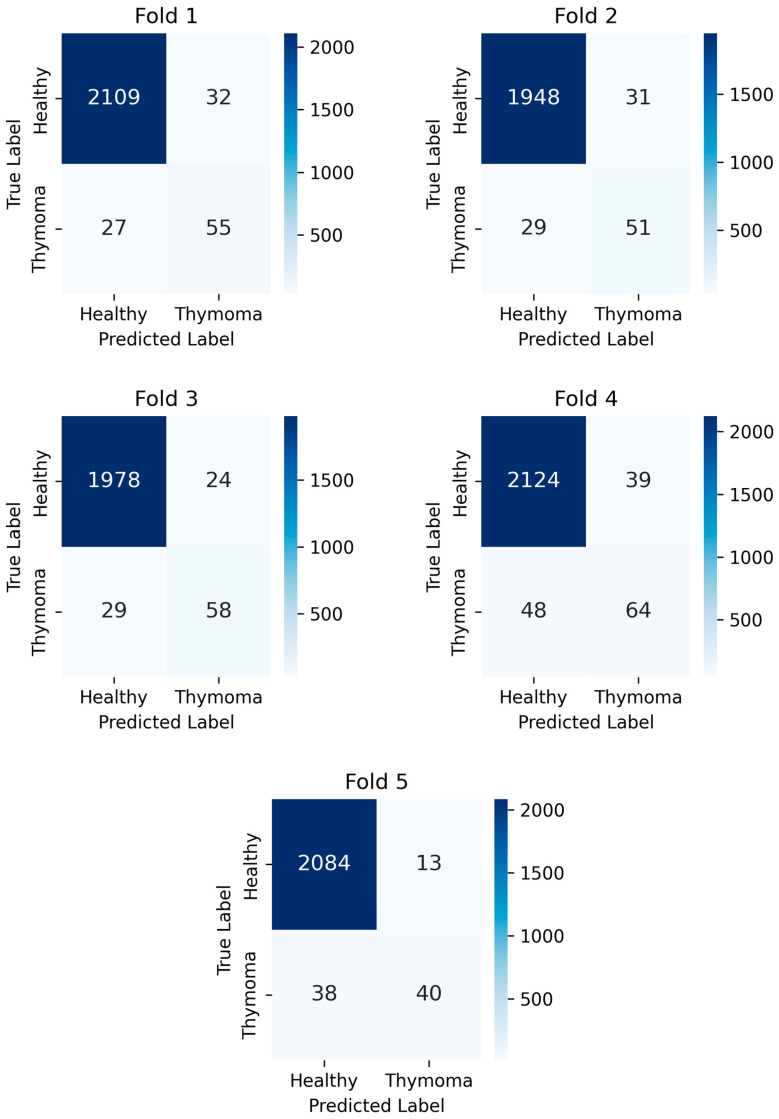
Confusion matrices of the proposed model for 5-fold cross-validation on thymoma-healthy classification.

In the binary classification task that distinguishes thymoma from healthy patients, 5-fold cross-validation was used to evaluate the effectiveness of the proposed model. The confusion matrices obtained for each fold are presented in Figure 6. In fold 1, the model correctly identifies 2109 out of 2141 healthy images and 55 out of 82 thymoma images. It misclassifies 32 healthy images as thymoma and 27 thymoma images as healthy. This balance in error distribution reflects that the model does not significantly favor one class over the other. With both precision and recall above 98% and an accuracy of 97.35%, the model demonstrates a very reliable performance for both image types in this fold. In fold 2, the model correctly classifies 1948 healthy images and 51 thymoma images. However, 31 healthy images are wrongly predicted as thymoma and 29 thymoma images are misclassified as healthy. This results in a nearly equal number of classification errors across the two classes. Despite these errors, the model maintains an impressive precision and recall above 98%, indicating reliable and stable performance in fold 2, with an overall accuracy of 97.09%. In fold 3, 1978 out of 2002 healthy images and 58 out of 87 thymoma images are correctly classified. There are 24 false negatives (healthy as thymoma) and 29 false positives (thymoma as healthy). This fold shows a slight increase in thymoma recognition accuracy compared to earlier folds, with the lowest number of misclassified healthy images so far. The resulting metrics, including F1-score of 98.63%, suggest balanced and high-quality predictions for both categories. In fold 4, the model correctly classifies 2124 healthy images and 64 thymoma images. However, 39 healthy images are mistakenly labeled as thymoma, and 48 thymoma images are misclassified as healthy. This fold has the highest number of misclassifications for both classes, particularly affecting the thymoma predictions. Despite this, the model still performs strongly, with a solid F1-score (97.99%) and an accuracy of 96.18%. The results hint that this fold may include more challenging or ambiguous thymoma cases. Fold 5 yields the highest recall (99.38%), with only 13 out of 2097 healthy images being misclassified. This demonstrates the model’s exceptional ability to detect healthy cases. However, 38 thymoma images are misclassified as healthy, reducing the precision slightly. Only 40 thymoma images are correctly identified. While the thymoma detection rate is lower, the overall classification results remain excellent, with an F1-score of nearly 98.8% and accuracy of 97.65%. This fold shows the model’s strength in avoiding false negatives for healthy, potentially at the expense of thymoma sensitivity.

#### 3.4.2. Classification Results of Benign and Malignant Thymomas

Table 2 provides a comparative performance evaluation of various DL models on the classification task of distinguishing benign from malignant thymoma using CT images. The metrics analyzed—accuracy, F1 Score, recall, and precision—offer a multidimensional perspective on each model’s diagnostic capability. Given the clinical significance of correctly identifying malignancies, this evaluation is crucial for reliable computer-aided diagnosis systems. Among the models tested, the proposed model clearly outperforms all others in every metric. The proposed model achieves an accuracy of 79.20%, which is the highest in the table, followed by VGG16 with 77.98% and InceptionV3 with 75.13%. These results indicate that the proposed model is more consistent in correctly identifying both benign and malignant cases compared to standard architectures. In terms of the F1 Score, which balances precision and recall and is especially important in imbalanced datasets or medical diagnostics, the proposed model again takes the lead with 78.51%. This high F1 score signifies that the model effectively identifies true tumor classes while minimizing false predictions. VGG16 and InceptionV3 also perform well in this metric (76.52% and 73.90%, respectively), but remain below the proposed model. When focusing on the recall, which measures the model’s ability to correctly detect actual malignant cases, the proposed model delivers 79.30%, slightly above InceptionV3 (78.01%) and VGG16 (77.55%). This suggests that the proposed model is less likely to miss malignant cases, a critical feature in medical diagnostics where false negatives can be dangerous. The precision, which measures the proportion of correct positive identifications, is once again highest in the proposed model at 79.88%. This demonstrates that it not only detects malignant cases effectively but also avoids misclassifying benign ones as malignant—an important aspect for preventing over-treatment or patient anxiety. In contrast, models like ResNet34 and ResNet50 perform poorly across all metrics, with accuracies around 64% and 63%, respectively, and F1 scores barely above 60%. These models struggle significantly in this classification task, suggesting their features may not be well suited for differentiating subtle variations in CT scans between benign and malignant thymoma. In conclusion, the proposed model exhibits superior performance across all evaluation metrics, making it a promising candidate model for practical deployment in clinical environments to support radiologists in classifying thymoma tumors. Its balanced and high scores suggest it can reliably aid in distinguishing between benign and malignant forms, thereby supporting timely and accurate medical decision-making.

**Figure 7:**
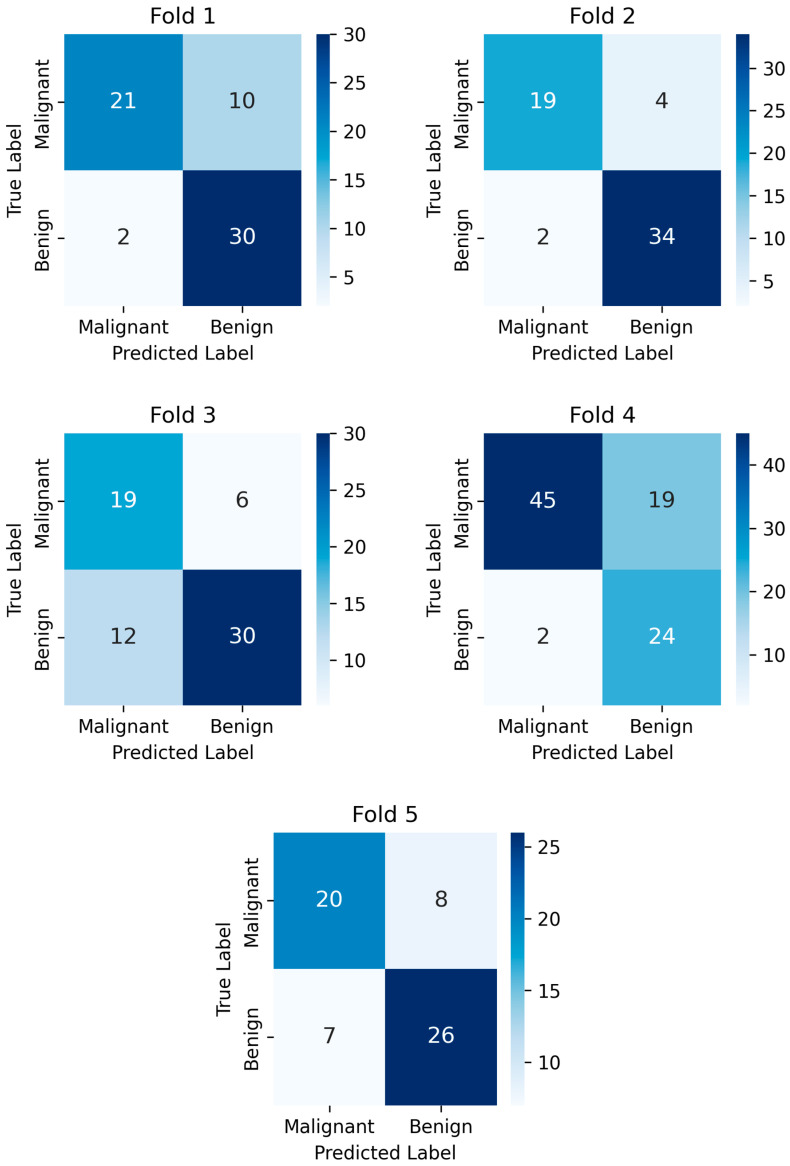
Confusion matrices of the proposed model for 5-fold cross-validation on benign—malignant thymoma classification.

Figure 7 presents the confusion matrices obtained from each fold during the 5-fold cross-validation process of the proposed model, conducted for the classification of malignant and benign thymoma tumors. In fold 1, the model correctly identified 21 malignant and 30 benign cases, while misclassifying 10 malignant as benign (false negatives) and 2 benign as malignant (false positives). The precision for malignant tumor detection, which evaluates the correctness of positive predictions, was calculated as approximately 91.3%, indicating that most of the samples predicted as malignant were indeed malignant. However, the recall—which reflects the model’s ability to identify actual malignant cases—was lower at 67.7%, due to the relatively high number of false negatives. This trade-off between precision and recall resulted in an F1 score of approximately 77.8%, representing the harmonic mean of both. The overall accuracy for fold 1 was about 81.0%, showing moderate general performance with a notable room for improvement in sensitivity (recall). In fold 2, the model achieved higher performance across all metrics. It correctly classified 19 malignant and 34 benign cases, with only 4 false negatives and 2 false positives. The precision reached approximately 90.5%, indicating high confidence in the malignant predictions. The recall improved compared to fold 1 and was calculated as 82.6%, showing better success in identifying actual malignant cases. These strong precision and recall values yielded an F1 score of about 86.4%. The overall accuracy was also higher, approximately 89.8%, indicating a balanced and reliable performance by the model in this fold. Fold 3 showed a noticeable decline in performance compared to fold 2. The model correctly predicted 19 malignant and 30 benign cases, but it misclassified 6 malignant tumors as benign and 12 benign tumors as malignant. This imbalance led to a precision of only 61.3%, meaning that a considerable number of the malignant predictions were incorrect. The recall, which measures the correct identification of malignant cases, was 76.0%. As a result, the F1 score dropped to 67.8%, reflecting the disparity between precision and recall. The overall accuracy was approximately 73.1%, the lowest among all folds. Fold 4 had a higher number of malignant cases, with the model successfully identifying 45 out of 64, but missing 19 (false negatives). It also correctly predicted 24 benign cases, with only 2 false positives. The precision was excellent at 95.7%, suggesting that nearly all malignant predictions were accurate. However, the recall was 70.3%, reflecting a considerable number of missed malignant tumors. Consequently, the F1 score balanced the two at approximately 81.2%. The overall accuracy was around 76.7%, which, although respectable, reveals the trade-off between high precision and limited recall in this fold. In fold 5, the model identified 20 malignant and 26 benign cases correctly, with 8 false negatives and 7 false positives. The precision was 74.1%, lower than previous folds, indicating more false alarms in malignant predictions. The recall was 71.4%, showing a moderate capacity to detect actual malignant cases. These values combined to yield an F1 score of 72.7%, signifying a balanced but modest performance. The accuracy for fold 5 was about 75.4%, indicating an average classification performance compared to the other folds.

### 3.5. Ablation Analysis

#### 3.5.1. Analysis of Model Components

First of all, the model component analysis, which includes the separate contributions of the VGG16 and MLP-Mixer models in the VGG16-MLP-Mixer model proposed for the classification of Thymoma and healthy cases in the ablation analysis, is given in Table 3. According to the table, both VGG16 and MLP-Mixer models individually contribute significantly to the classification performance, with VGG16 achieving 96.22% accuracy and F1 score of 75.57%, while MLP-Mixer achieves slightly lower performance with 95.75% accuracy and F1 score of 66.73%. When these two components are combined, the proposed VGG16-MLP-Mixer model reaches the highest performance, with 97.15% accuracy and F1 score of 80.99%. This indicates that the hybrid model benefits from the complementary strengths of both architectures, leading to improved overall classification performance. Notably, the increase in the F1 score in the combined model suggests a more balanced improvement in precision and recall, which is particularly important for medical classification tasks where both false positives and false negatives carry significant consequences.

#### 3.5.2. Different Backbone Networks in MLP-Mixer Model

Subsequently, experimental studies were carried out by replacing the VGG16 backbone in the MLP-Mixer model with various alternative backbone networks to evaluate their impact on classification performance, and the resulting performance metrics are presented in Table 4. This table provides a comparative evaluation of backbone networks—InceptionV3, DenseNet121, ResNet50, MobileNetV2, and VGG16—integrated into the MLP-Mixer model for classifying Thymoma and healthy cases. Among these, the VGG16 backbone achieved the best overall performance, with the highest accuracy (97.15%), F1 score (80.99%), recall (82.56%), and precision (79.92%). DenseNet121 followed closely, particularly in recall (82.13%) and F1 score (77.40%), but fell short of VGG16’s overall balance. InceptionV3 and ResNet50 produced comparable results, with respectable accuracy and F1 scores around 75%, though with slightly lower precision and recall. MobileNetV2 demonstrated the weakest performance across all metrics, especially in accuracy (92.26%) and recall (74.17%), indicating limited suitability for this task. Overall, the experimental results in Table 4 clearly demonstrate that VGG16 is the most effective backbone network when paired with the MLP-Mixer, outperforming all alternatives in every key metric. This reinforces the selection of VGG16 in the proposed model and highlights its successful feature extraction capability for the classification of thymoma versus healthy cases.

#### 3.5.3. Preprocessing Stage Analysis

To evaluate the effectiveness of using cropped images in the preprocessing step, the proposed model was tested in an ablation analysis alongside various DL-based models. In this analysis, experimental studies were conducted separately for single-channel and RGB inputs, and a comparison was made with the use of entire slice images to determine the impact of cropping. The F1 score values resulting from these experiments are presented in Table 5. Table 5 presents the F1 scores achieved by different models—InceptionResNetV2, DenseNet121, InceptionV3, VGG16, and the proposed model—under two preprocessing scenarios: entire slice image and cropped image, each evaluated with both single-channel and RGB formats. The results clearly show that the proposed model consistently outperforms the others across all preprocessing types. Notably, it achieves the highest F1 score (80.99%) when using cropped images in RGB format, confirming the effectiveness of both the cropping strategy and color information in enhancing classification performance. Comparatively, all models show improved performance when using cropped images versus entire slices, especially with RGB inputs—e.g., VGG16 improves from 74.53% to 75.57%, and InceptionResNetV2 from 72.30% to 76.83%. This trend highlights the advantage of focusing on the region of interest in medical imaging. Overall, these findings validate the preprocessing choice in the proposed model and demonstrate that both cropping and RGB formatting contribute significantly to improving model performance.

#### 3.5.4. Aggregation of Slices

Finally, instead of working on individual slices separately, all slices were combined and experimental studies were conducted accordingly. The results obtained using the proposed model are presented in Table 6. Table 6 shows that after scanning all CT slices, if at least one slice contains a thymoma, the corresponding CT image is labeled as belonging to a patient with thymoma. Upon analyzing the 5-fold cross-validation results of the proposed model, it is observed that the true positive (TP—healthy) and true negative (TN—thymoma) rates are generally high. On average, 17.2 individuals were correctly classified as healthy, while 15 individuals were accurately labeled as having thymoma. This indicates that the model demonstrates strong performance in both identifying healthy individuals and detecting patients with thymoma, highlighting its potential reliability for clinical decision-making. The false positive and false negative rates remain limited. On average, 6.8 individuals were incorrectly classified as having thymoma, while 0.6 thymoma patients were misclassified as healthy. The low number of false negatives indicates that the likelihood of missing thymoma patients is minimal, demonstrating a critical strength of the model. However, the false positive rate is relatively high in some folds (e.g., fold 4–11 false positives), suggesting that the model occasionally labels healthy individuals as at risk unnecessarily. This is an important consideration in terms of further examinations and patient psychology. Overall, the model performs in a balanced manner and produces clinically meaningful results. The especially low false negative rate demonstrates the model’s ability to detect thymoma patients without missing cases, while high true negative values provide a significant advantage in accurately identifying affected individuals. However, reducing the false positive rate could improve the model’s specificity, lower the number of false alarms, and thus help reduce unnecessary burdens on the healthcare system. In this context, additional optimization studies focusing on minimizing false positives could help further refine the model. In the results above, except for fold 5, all thymoma patients were correctly classified in the other folds. On the other hand, in fold 5, three thymoma patients were incorrectly labeled as healthy.

### 3.6. GRAD-CAM Analysis

**Figure 8:**
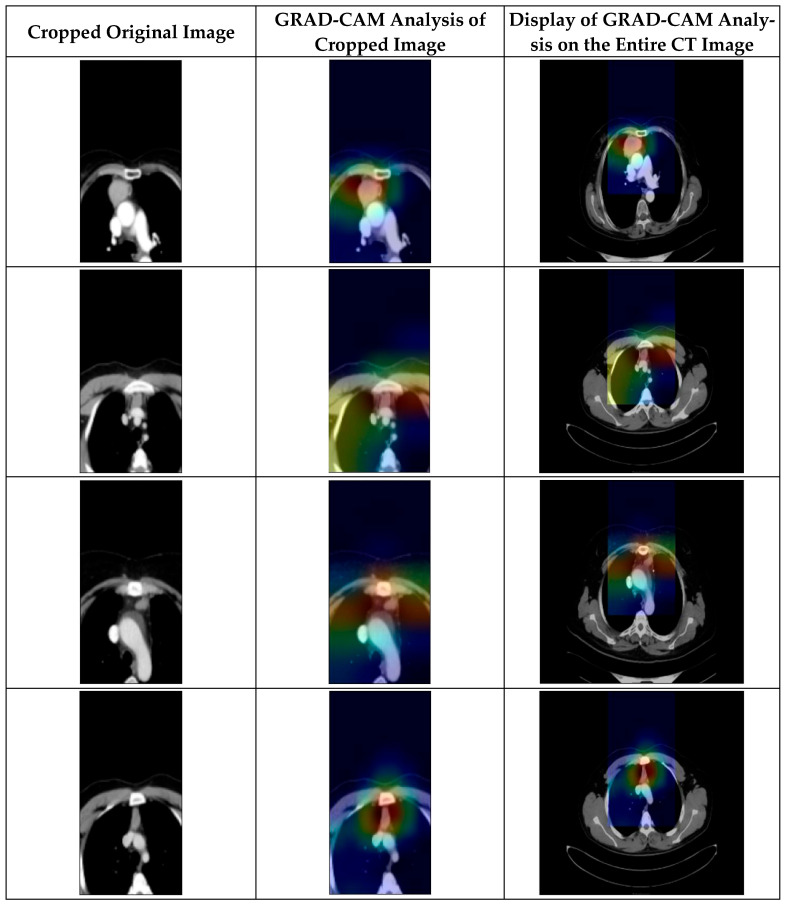
GRAD-CAM analysis of CT images of samples with thymoma tumors.

Some examples of CT images containing thymoma tumors are shown in Figure 8. In the first column of Figure 8, the cropped original CT images of patients with thymoma tumors are displayed. These cropped images are obtained by focusing specifically on the region of interest in the mediastinal area where the tumor is located. By removing irrelevant surrounding anatomical structures, the cropped images highlight only the potential tumor region. This preprocessing step allows the model to concentrate on significant pathological features while minimizing background noise, thereby improving both detection accuracy and interpretability of the subsequent analysis. In the second column, the Gradient-weighted Class Activation Mapping (GRAD-CAM) [32] analysis is applied to the cropped images to visualize the regions that most influenced the model’s decision. The color spectrum in these heatmaps, ranging from blue to red, represents the relative importance of different regions, with red indicating areas of highest model attention. Across all samples, it is evident that the red zones are concentrated around the suspected tumor regions, demonstrating that the model effectively identifies and focuses on diagnostically significant areas. The highlighted zones consistently align with the suspected tumor masses in the mediastinal region, demonstrating that the model effectively captures discriminative features related to thymoma presence rather than focusing on irrelevant artifacts. The third column displays the same GRAD-CAM activations from the cropped image analysis, this time overlaid on the full, uncropped CT images. This visualization offers a comprehensive spatial context by showing exactly where the model’s attention falls within the entire thoracic cavity. In each case, the highlighted heatmap regions correctly correspond to the anatomical location of the tumor, reinforcing the consistency and reliability of the model’s focus. By presenting both localized (cropped) and holistic (entire image) interpretations, Figure 8 provides strong visual evidence of the model’s ability to detect thymoma tumors in a clinically meaningful and interpretable way.

### 3.7. Limitations

This study has several limitations that should be acknowledged. First, the dataset used in this work originates from a single institution. Although substantial effort was devoted to data preparation, the rarity of thymoma poses significant challenges for multicenter data collection, and assembling a larger, externally diverse cohort was not feasible within the scope of this study. As a result, the generalizability of the proposed deep learning model may be limited, and caution should be exercised when extending these findings to broader clinical populations. Second, due to the limited availability of data for this rare disease, an independent external validation set could not be obtained. While we employed a rigorous cross-validation strategy to enhance robustness and mitigate overfitting, external validation remains an important step for future work to better assess the model’s performance across institutions, imaging protocols, and patient demographics. Lastly, although the model demonstrated promising results, further evaluation on larger and more heterogeneous datasets—including multicenter studies—will be necessary to confirm its clinical applicability and effectiveness in real-world settings.

## 4. Conclusions and Future Prospects

This study introduces a novel DL model that synergistically combines a pre-trained VGG16 backbone with an MLP-Mixer-based feature enhancement module for the classification of thymoma tumors using CT imaging. Experimental evaluations demonstrate that the proposed model outperforms several state-of-the-art architectures—including SEResNeXt50, MobileNetV2, InceptionV3, InceptionResNetV2, DenseNet121, ResNet34, and ResNet50—across two critical clinical classification tasks: distinguishing thymoma from healthy tissue and differentiating benign from malignant thymoma. For the thymoma versus healthy classification, the proposed model achieved the highest accuracy of 97.15%, surpassing the closest competitors InceptionResNetV2 (97.02%) and ResNet34 (97.07%). It also led in F1 score (80.99%) and precision (79.92%), demonstrating a superior balance between recall and precision—key factors in reducing false negatives and false positives in clinical practice. Although ResNet34 showed the highest recall (85.99%), its lower precision resulted in a diminished overall F1 score, highlighting the importance of balanced performance metrics in medical diagnostics. In the more challenging benign versus malignant thymoma classification, the proposed model again attained the highest accuracy of 79.20%, with F1 score of 78.51%, recall of 79.30%, and precision of 79.88%. These results indicate the model’s enhanced capability in identifying malignancies accurately while minimizing misclassification of benign cases. Conventional architectures such as ResNet34 and ResNet50 struggled significantly on this task, emphasizing the effectiveness of the combined VGG16 and MLP-Mixer approach in capturing subtle radiological differences indicative of malignancy. These findings underscore the model’s potential as a valuable tool for aiding radiologists in timely and accurate thymoma diagnosis, ultimately contributing to improved patient management and treatment planning. Overall, the proposed model demonstrates robust and consistent performance across multiple evaluation metrics, suggesting its potential as a reliable tool for clinical decision support in thymoma diagnosis. The integration of VGG16’s powerful spatial feature extraction with the MLP-Mixer’s global feature modeling enables improved classification accuracy and generalization, advancing the field of automated thoracic tumor analysis. In future studies, the primary focus will be on developing different deep learning models to improve the classification performance between benign and malignant thymoma. Additionally, research efforts will aim to extend the proposed framework to the segmentation of thymoma tumors in CT images. Accurate tumor segmentation is critical for precise localization, volumetric analysis, and treatment planning, thereby enhancing the clinical utility of automated diagnostic systems. By integrating advanced segmentation techniques with the current classification model, subsequent studies intend to develop a comprehensive computer-aided diagnosis tool that supports both detection and characterization of thymoma tumors. This approach is expected to significantly contribute to improved patient management and treatment outcomes.

## Figures and Tables

**Table 1:** Performance Comparison of DL Models for Thymoma/Healthy Classification.

Models	Accuracy (%)	F1 Score (%)	Recall (%)	Precision (%)
SEResNeXt50	96.83	75.57	81.82	72.47
MobileNetV2	96.63	74.92	80.22	72.68
InceptionV3	96.88	75.53	82.71	71.60
InceptionResNetV2	97.02	76.83	83.83	72.55
DenseNet121	96.98	77.51	82.65	74.01
VGG 16	96.22	75.57	79.39	74.56
ResNet34	97.07	75.64	85.99	71.72
ResNet50	96.88	76.94	81.27	73.82
ViT	78.75	60.28	73.19	57.48
Swin Transformer	84.52	66.46	75.38	63.13
Proposed Model	97.15	80.99	82.56	79.92

**Table 2:** Comparison of DL Models for Benign—Malignant Thymoma Classification.

Models	Accuracy (%)	F1 Score (%)	Recall (%)	Precision (%)
SEResNeXt50	71.59	70.16	73.65	72.40
MobileNetV2	66.36	65.59	70.22	68.80
InceptionV3	75.13	73.90	78.01	75.59
InceptionResNetV2	73.11	71.74	73.28	72.39
DenseNet121	70.91	69.70	70.19	71.22
VGG 16	77.98	76.52	77.55	76.78
ResNet34	64.30	62.67	72.64	67.98
ResNet50	63.36	60.66	72.02	67.42
ViT	44.56	41.29	47.32	43.84
Swin Transformer	54.71	52.47	58.12	54.98
Proposed Model	79.20	78.51	79.30	79.88

**Table 3:** Contribution Analysis of Model Components.

VGG16	MLP Mixer	Accuracy (%)	F1 Score (%)
X		96.22	75.57
	X	95.75	66.73
X	X	97.15	80.99

**Table 4:** Comparison of Different Backbone Networks in MLP-Mixer Model.

Models	Accuracy (%)	F1 Score (%)	Recall (%)	Precision (%)
InceptionV3	96.90	75.46	82.59	72.18
DenseNet121	96.93	77.40	82.13	74.05
ResNet50	96.78	75.07	81.16	71.43
MobileNetV2	92.26	71.86	74.17	74.27
VGG16	97.15	80.99	82.56	79.92

**Table 5:** F1 Scores Resulting from Preprocessing Stage Analysis (%).

Models	Entire Slice Image		Cropped Image	
	Single Channel	RGB	Single Channel	RGB
InceptionResNetV2	72.31	72.30	72.19	76.83
DenseNet121	70.43	76.56	76.51	77.51
InceptionV3	70.56	74.47	76.47	75.53
VGG16	73.71	74.53	73.73	75.57
Proposed Model	73.54	77.90	77.59	80.99

**Table 6:** Results from the Aggregation of Slices.

Fold	Accuracy (%)	F1 Score (%)	Precision (%)	Recall (%)	TP	FP	FN	TN
Fold 1	87.18	87.15	89.13	88.10	18	5	0	16
Fold 2	82.50	82.40	86.00	84.09	18	7	0	15
Fold 3	82.50	82.49	85.42	84.78	17	7	0	16
Fold 4	72.50	72.34	77.08	79.63	13	11	0	16
Fold 5	82.05	81.26	81.67	80.98	20	4	3	12
Average Results	81.35	81.13	83.86	83.52	17.2	6.8	0.6	15

## Data Availability

The data presented in this study are available on request from the corresponding author. The data are not publicly available due to privacy reasons.

## Abbreviations

The following abbreviations are used in this manuscript:

CT	Computed tomography
AI	Artificial intelligence
DL	Deep learning
MRI	Magnetic resonance imaging
GRAD-CAM	Gradient-weighted Class Activation Mapping
ROI	Regions of interest
MLP	Multilayer perceptron
TP	True positives
TN	True negatives
FN	False negatives
FP	False positives
GELU	Gaussian error linear unit
ViT	Vision Transformer
GAP	Global average pooling
